# Willingness to repeat discharge on day of surgery after hip and knee arthroplasty

**DOI:** 10.1302/2633-1462.69.BJO-2025-0109.R1

**Published:** 2025-09-26

**Authors:** Oddrún Danielsen, Kirill Gromov, Claus Varnum, Thomas H. Jakobsen, Mikkel R. Andersen, Manuel J. Bieder, Christoffer C. Jørgensen, Henrik Kehlet, Martin Lindberg-Larsen

**Affiliations:** 1 Center for Fast-track Hip and Knee Replacement, Aalborg, Denmark; 2 Department of Orthopaedic Surgery and Traumatology, Odense University Hospital and Svendborg, Odense, Denmark; 3 Department of Orthopaedic Surgery, Hvidovre University Hospital, Hvidovre, Denmark; 4 Department of Orthopaedic Surgery, Lillebaelt Hospital – Vejle, Vejle, Denmark; 5 Department of Orthopaedic Surgery, Aalborg University Hospital, Aalborg, Denmark; 6 Department of Orthopaedic Surgery, Copenhagen University Hospital, Herlev-Gentofte, Denmark; 7 Department of Orthopaedic Surgery, Næstved, Slagelse and Ringsted Hospitals, Slagelse, Denmark; 8 Department of Anaesthesia, Hospital of Northern Zeeland, Hillerød, Denmark; 9 Section of Surgical Pathophysiology, Copenhagen University Hospital, Rigshospitalet, Copenhagen, Denmark

**Keywords:** Hip arthroplasty, Knee arthroplasty, Fast-track, Day-case surgery, Same day discharge, hip and knee arthroplasties, knee arthroplasty procedures, Hip, joint arthroplasty, total knee arthroplasty (TKA), medial unicompartmental knee arthroplasty, primary total hip arthroplasty, cohort study, total hip arthroplasty, fast

## Abstract

**Aims:**

The limited documentation on patients’ perspectives on undergoing discharge on the day of surgery impedes its adoption as a standard of care. Hence, the aim of this study was to investigate whether patients were willing to repeat being discharged on the day of surgery if having a future hip or knee arthroplasty procedure.

**Methods:**

This multicentre, prospective consecutive cohort study spanned from 1 September 2022 to 31 January 2024, and was conducted at six public arthroplasty centres adhering to the same published protocol for discharge on the day of surgery following hip and knee arthroplasty. Patients undergoing primary total hip arthroplasty (THA), total knee arthroplasty (TKA), or medial unicompartmental knee arthroplasty (mUKA) were screened for eligibility and discharged when fulfilling predetermined discharge criteria. Patients discharged on the same calendar day of surgery were sent a questionnaire 30 days postoperatively.

**Results:**

Of 9,542 primary hip and knee arthroplasties registered, 3,457 (36%) were eligible for discharge on day of surgery; 58% of eligible patients (n = 2,011) were discharged on day of surgery and therefore received the survey. Baseline characteristics were comparable across all arthroplasty groups. The survey response rate was 88% (n = 1,771). Overall, 90% (95% CI 88 to 91) were willing to repeat discharge on the day of surgery if having a future joint arthroplasty, with 91% (95% CI 88 to 93) after THA, 89% (95% CI 86 to 92) after TKA, and 90% (95% CI 86 to 92) after mUKA. The difference between centres ranged from 84% to 93%. Patients responding ‘no’ to repeat discharge on the day of surgery were more often female (55%, n = 95) compared to patients responding ‘yes’ (47%, n = 744); otherwise, the groups were comparable.

**Conclusion:**

A total of 90% of patients (n = 1,590) discharged on the day of surgery following hip and knee arthroplasty expressed willingness to repeat discharge on the day of surgery. This supports further implementation efforts.

Cite this article: *Bone Jt Open* 2025;6(9):1156–1163.

## Introduction

With the anticipated rise in hip and knee arthroplasty demand due to an ageing and increasingly active population, optimizing the length of hospital stay while ensuring patient safety remains a key focus. This has led to the increased use of fast-track/enhanced recovery protocols in recent years,^1-3^ also serving as a cost-saving effort.^4^ The ultimate goal of fast-track surgery may be to discharge patients on the day of surgery without compromising safety and satisfaction.^5,6^ The feasibility of implementing discharge on the day of surgery in 20% to 25% of all primary hip and knee arthroplasty patients in a public multicentre setting, representing 40% of a Danish nationwide production, is now well established.^7,8^ However, despite the growing acceptance of discharging patients on the day of surgery, there is a paucity of research dealing with patients’ perspectives on discharge on the day of surgery for these procedures.^9^

While the clinical efficacy of discharge on the day of surgery is well established, the successful implementation of discharge on the day of surgery depends on more than just medical outcomes—it necessitates a comprehensive understanding of patients’ experiences and preferences. Without thorough documentation of patients’ perspectives, it becomes challenging to advocate for the widespread adoption of discharge on the day of surgery as a standard of care.

Hence, the aim of the study was to investigate whether patients were willing to repeat being discharged on the day of surgery if they were to have a future hip or knee arthroplasty procedure.

## Methods

### Study design

This was a prospective cohort study following the REporting of studies Conducted using Observational Routinely-collected Data (RECORD) guideline.^10^

### Setting

**Fig. 1:**
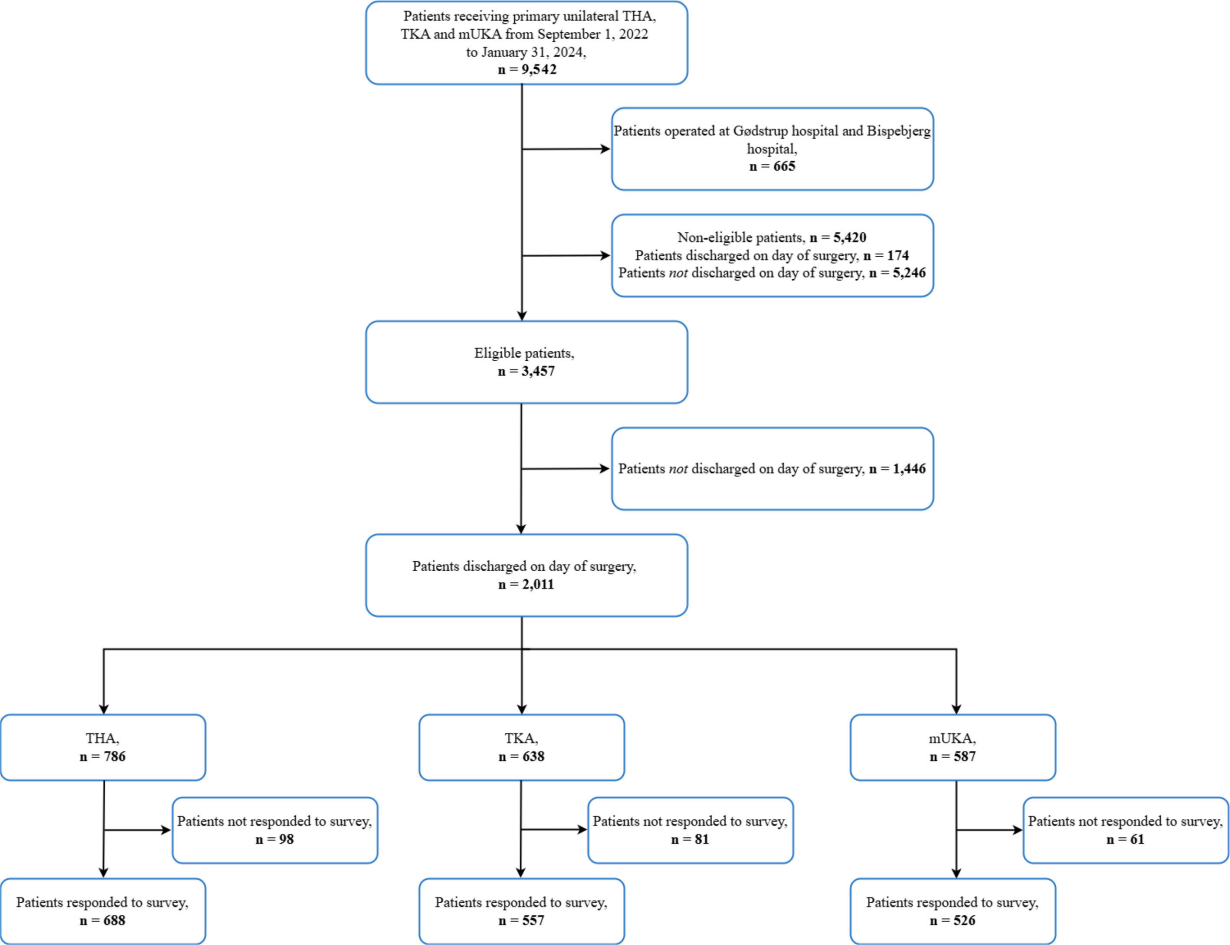
Flowchart of patient inclusion in the study. THA, total hip arthroplasty; TKA, total knee arthroplasty; mUKA, medial unicompartmental knee arthroplasty

This study emanated from the multicentre collaboration ‘The Center for Fast-track Hip and Knee Replacement’.^11^ The collaboration consists of eight public arthroplasty centres covering 40% of a Danish nationwide production of hip and knee arthroplasty procedures. For this study, we only included the six centres that maintained a well-established protocol for discharge on the day of surgery throughout the study period.^7^ Hence, one centre in the collaboration was excluded as they did not manage to implement the protocol sufficiently during the study period (33 patients discharged on the day of surgery in the study period). Another centre was excluded due to a delayed start of inclusion caused by relocation to a new hospital site. The study period was from 1 September 2022 to 31 January 2024 (Figure 1).

### Study population

The cohort included patients undergoing primary total hip arthroplasty (THA), total knee arthroplasty (TKA), and medial unicompartmental knee arthroplasty (mUKA) from a multicentre healthcare setting receiving patients who had not been preselected. The protocol for discharge on the day of surgery within the collaboration has been published.^7^ Patients’ eligibility for discharge on the day of surgery was evaluated using clearly specified inclusion and exclusion criteria (Supplementary Material). Patients were discharged upon meeting the predetermined discharge criteria (Supplementary Material). For this study, we only included eligible patients who were discharged on the same calendar day as surgery (Figure 1). Some of the patients included in this study cohort have also been reported on in previous publications, but with a shorter study period and a different endpoint.^8,12^

### Data sources

At each centre, dedicated research staff prospectively collected patient data, with the option of physician assistance if necessary. The data were securely stored in an online REDCap database provided by the Open Patient Data Explorative Network (OPEN) at Odense University Hospital. The database comprised patient-reported data as well as information extracted from patient files.^7^ Eligible patients discharged on day of surgery received a survey 30 days postoperatively with the question: ‘If you were to undergo hip/knee arthroplasty on the opposite side, would you prefer to be discharged on the day of surgery again?’.

### Variables

Demographic variables were age (continuous variable), sex (male/female), BMI (kg/m^2^), cohabitation (cohabiting, living alone), Clinical Frailty Scale (1 to 4),^13^ alcohol consumption above ten units (yes/no), regular home assistance before surgery (yes/no), previous cerebrovascular accident (yes/no), preoperative psychiatric medication (yes/no), and preoperative opioid use (yes/no).

The primary outcome variable was the proportion of patients discharged on the day of surgery who were willing to repeat being discharged on the day of surgery if undergoing a second hip or knee arthroplasty procedure. This outcome variable was analyzed overall as well as by procedure and centre level. Secondary outcome variables were differences in baseline characteristics between ‘yes’ responders and ‘no’ responders” (Table I). An additional subanalysis also compared ‘responders’ and ‘non responders’ (Supplementary Material).

### Ethics and registration

Patients were included after informed consent. Since the outpatient surgical treatment for eligible patients adhered to the standard of care at the collaborating centres, as specified in the protocol,^7^ ethical approval was deemed unnecessary according to Danish law.

The fast-track project was preregistered at ClinicalTrials.gov (NCT05613439) and within the Region of Southern Denmark, with the necessary data processing approval obtained (Journal No. 22/39454).

**Table I.** Characteristics of responders.

Variable	‘Yes’ responders n = 1,590	‘No’ responders n = 174
**Mean age, yrs (SD)**	66.0 (8.5)	65.4 (8.7)
**Sex, n (%)**		
Female	744 (47)	95 (55)
Male	835 (53)	77 (44)
Missing	11 (0.7)	2 (1.1)
**Mean BMI, kg/m** ^**2**^ **(SD)**	28.9 (4.5)	27 (4.3)
**Cohabitation, n (%)**		
Cohabiting	1,404 (88)	160 (92)
Living alone	176 (11)	12 (7)
Missing	10 (0.6)	2 (1.1)
**Mean CFS (SD)**	2.2 (0.8)	2.2 (0.7)
**Alcohol consumption above 10 units, n (%)**	254 (16)	27 (16)
**Regular home assistance before surgery, n (%)**	10 (0.6)	2 (1)
**Previous cerebral accident, n (%)**	67 (4)	7 (4)
**Preoperative psychiatric medication, n (%)**		
Yes	106 (7)	16 (9)
No	1,477 (93)	158 (91)
Missing	7 (0.4)	0
**Preoperative opioid use, n (%)**		
Yes	62 (4)	10 (6)
No	1524 (96)	164 (94)
Missing	4 (0.3)	0

CFS, Clinical Frailty Scale.

### Statistical analysis

Descriptive statistics were presented as means with SDs for continuous variables that followed a normal distribution. For non-normally distributed continuous variables, medians along with IQRs were presented. Categorical variables were described as proportions with 95% CIs and analyzed using Stata Statistical Software v. 18 (StataCorp, USA).

## Results

Among the 9,542 primary hip and knee arthroplasties registered in the database, 3,457 (36%) were eligible for discharge on the day of surgery. Overall, 58% of eligible patients (n = 2,011) were discharged on the day of surgery and hence 2,011 patients received the survey: 786 (39%) THA, 638 (32%) TKA, and 587 mUKA (29%) (Figure 1).

**Fig. 2:**
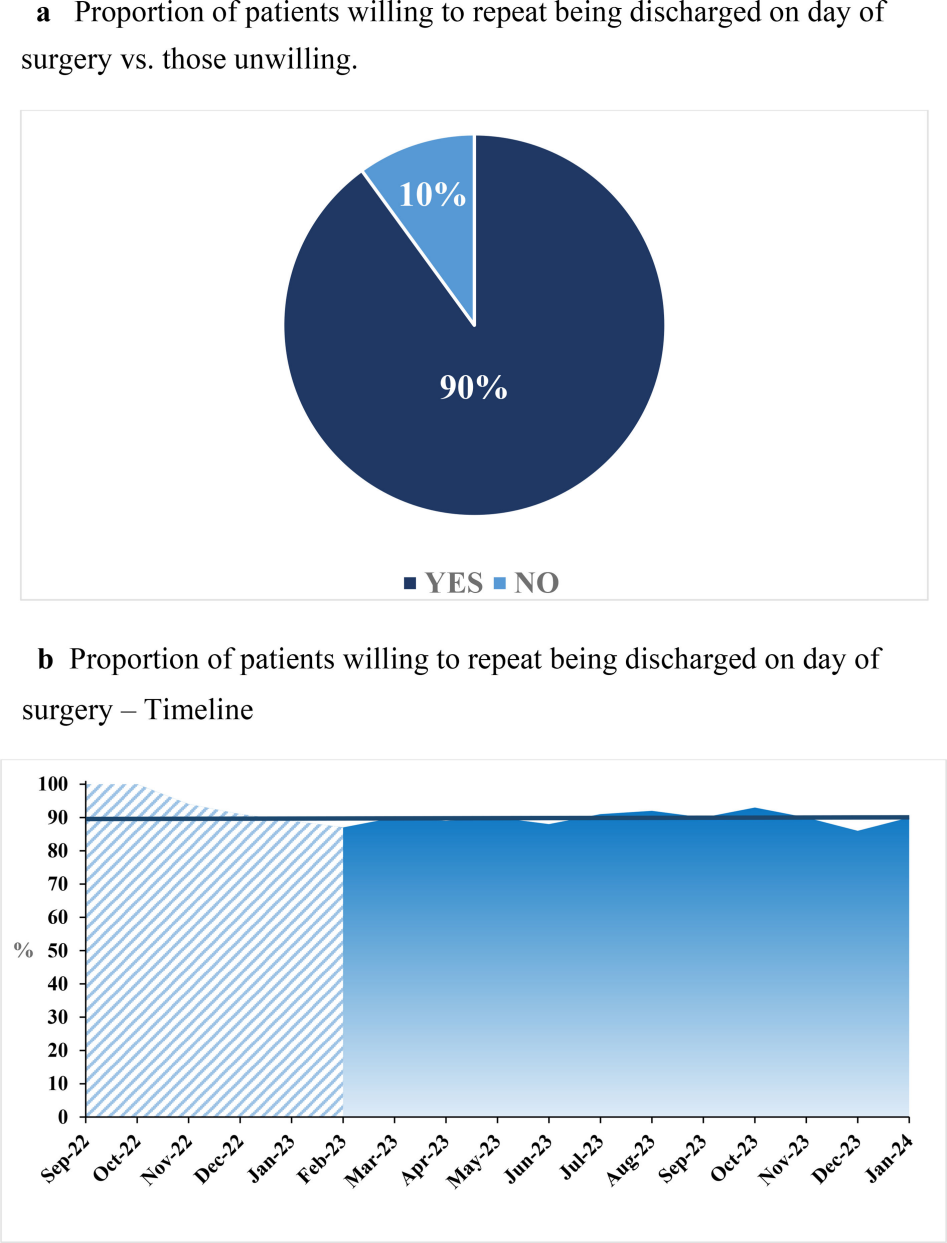
a) Proportion of patients willing to repeat being discharged on day of surgery compared with those unwilling. b) Proportion of patients willing to repeat being discharged on day of surgery, presented on a timeline.

**Fig. 3:**
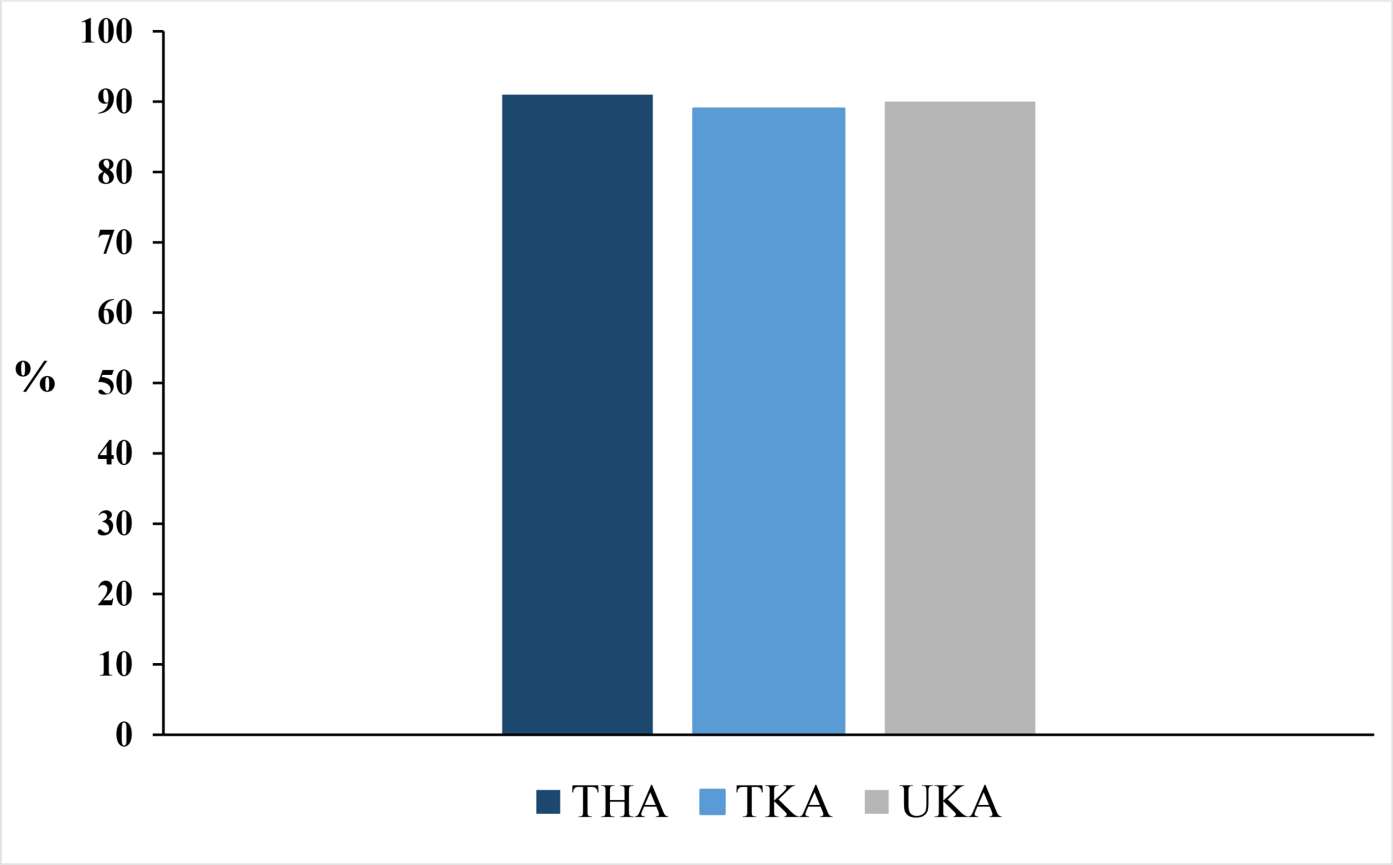
Proportion of patients willing to repeat being discharged on day of surgery divided by surgical procedure. THA, total hip arthroplasty; TKA, total knee arthroplasty; UKA, unicompartmental knee arthroplasty.

**Fig. 4:**
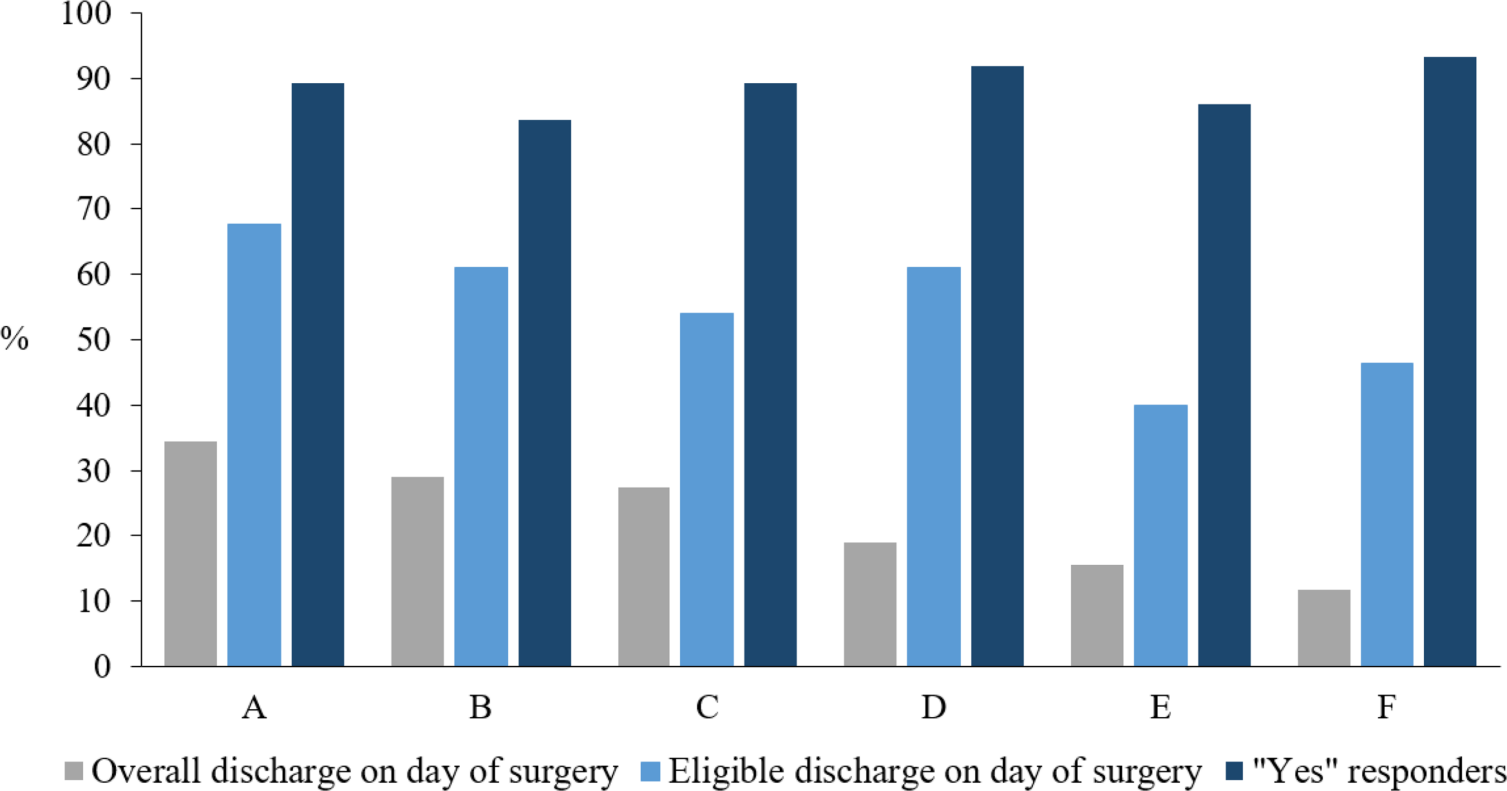
Proportion of patients willing to repeat being discharged on the day of surgery divided by centre. Overall discharge on the day of surgery represents the proportion of eligible patients discharged on the day of surgery out of all patients. Eligible discharge on day of surgery represents the proportion of eligible patients discharged on day of surgery out of eligible patients. Proportion of 'yes' responders.

Baseline characteristics were comparable across all three arthroplasty groups (Table II). The overall survey response rate was 88% (n = 1,771). In total, 90% (95% CI 88 to 91) of the respondents were willing to repeat being discharged on the day of surgery, if they were to have a second hip or knee arthroplasty procedure. The proportion of patients willing to repeat being discharged on the day of surgery remained consistent throughout the study period (Figure 2b). Procedure-specific willingness to repeat discharge on the day of surgery was 91% (95% CI 88 to 93) after THA, 89% (95% CI 86 to 92) after TKA, and 90% (95% CI 86 to 92) after mUKA (Figure 3). The proportion of ‘yes’ responders varied between centres (84% to 93%) (Figure 4). Patients not willing to repeat discharge on the day of surgery were more often female (55%, n = 95) compared with patients willing to repeat discharge on the day of surgery (47%, n = 744). Otherwise, the groups were comparable. Additionally, a sensitivity analysis examining differences between age groups stratified by decades was performed, but no notable differences were identified. A greater proportion of patients lived alone among non-responders (78%, n = 187) compared to responders (9%, n = 163) (Supplementary Material).^14^

**Table II.** Patient demographic characteristics of all responders.

Variable	THA (n = 688)	TKA (n = 557)	mUKA (n = 526)
**Mean age, yrs (SD)**	65.0 (9.1)	66.1 (8.2)	65.5 (8.2)
**Sex, n (%)**			
Female	330 (48)	273 (49)	237 (45)
Male	355 (52)	276 (50)	287 (55)
Missing	3 (0.4)	8 (1.4)	2 (0.4)
**Mean BMI, kg/m** ^**2**^ **(SD)**	27.2 (4.2)	29.5 (4.5)	29.7 (4.4)
**Cohabitation, n (%)**			
Cohabiting	616 (90)	524 (94)	456 (87)
Living alone	68 (10)	29 (5)	66 (13)
Missing	4 (0.6)	4 (0.7)	4 (0.8)
**Mean CFS (SD)**	2.2 (0.8)	2.2 (0.8)	2.1 (0.8)
**Alcohol consumption above 10 units, n (%)**	117 (17)	86 (15)	79 (15)
**Regular home assistance before surgery, n (%)**	2 (0.3)	4 (0.7)	4 (0.8)
**Previous cerebral accident, n (%)**	18 (3)	24 (4)	30 (6)
**Preoperative psychiatric medication, n (%)**			
Yes	31 (5)	17 (3)	19 (4)
No	654 (95)	538 (97)	505 (96)
Missing	3 (0.4)	2 (0.4)	2 (0.4)
**Preoperative opioid use, n (%)**			
Yes	35 (5)	34 (6)	21 (4)
No	651 (95)	523 (94)	503 (96)
Missing	2 (0.3)	-	2 (0.4)

CFS, Clinical Frailty Scale; mUKA, medial unicompartmental knee arthroplasty; THA, total hip arthroplasty; TKA, total knee arthroplasty.

## Discussion

In this prospective multicentre cohort study, 90% of patients discharged on the day of surgery following primary unilateral hip or knee arthroplasty expressed their willingness to repeat being discharged on the day of surgery if undergoing a future hip or knee arthroplasty procedure. Assessing patients’ willingness to repeat being discharged on the day of surgery serves as a proxy for their satisfaction with the fast-track setup, as it is presumed that patients would not wish to repeat a course of treatment they found unsatisfactory.

Discharge on the day of surgery following hip or knee arthroplasty has generated increasing attention in recent years, especially after the COVID-19 pandemic. Nevertheless, patients’ perspectives on discharge on the day of surgery for these types of procedures remain less known. In a previous single-centre study from the USA,^15^ patients received a questionnaire prior to hip or knee arthroplasty: 32% of the patients reported feeling either uncomfortable or very uncomfortable being discharged within 23 hours after surgery. In another Danish single-centre study from one of the participating centres prior to the current multicentre collaboration, patients’ attitudes towards discharge on the day of surgery were examined preoperatively, when they were scheduled for surgery.^16^ It was found that only 42% were interested in discharge on the day of surgery. These findings were concerning just before the broader multicentre implementation of discharge on the day of surgery in Denmark. However, our results, which show a 90% willingness to repeat discharge on the day of surgery, alleviate these concerns. The variability in these findings can likely be attributed to the fact that the patients in the study by Halken et al^16^ were not preselected, and were asked before undergoing surgery without receiving any structured information about day-case surgery before answering the questionnaires. In contrast, the patients in our study answered the questionnaire after the arthroplasty procedure, and they were selected for discharge on the day of surgery based on well-defined criteria.

Lovasz et al^17^ presented findings with an even higher satisfaction rate than we report, with 98% of patients selected for discharge on the day of surgery expressing their willingness to repeat same-day discharge if they were to undergo a similar procedure. However, this study was a single-centre study with only 200 patients. Additionally, data collection for this study occurred during a six-week postoperative follow-up visit at the hospital, raising the possibility that some patients may have felt pressured to express satisfaction with their experience.

According to Figure 4, there was a variation in overall discharge on the day of surgery success rates among the participating centres, as well as differences in the proportion of ‘yes’ responders. However, there did not appear to be any obvious correlation between these two variables, suggesting that a higher proportion of patients discharged on the day of surgery did not necessarily lead to more or less satisfied patients.

The strength of our study primarily lies in its comprehensive and well-defined protocol for discharge on the day of surgery after hip and knee arthroplasty.^7^ ‘The Center for Fast-track Hip and Knee Replacement’ consists of public arthroplasty centres across all regions in Denmark and accounts for approximately 40% of all hip and knee arthroplasties in the country. This setup, combined with a high response rate (88%) and the largest cohort reported on so far, enhances the potential generalizability of our findings. Furthermore, we specifically included centres in this study that maintained a well-established protocol for discharge on the day of surgery. As standard of care, all patients eligible for discharge on the day of surgery were provided with preoperative information regarding the discharged plan, and their expectations were carefully managed.^7^ This also contributed to a high level of willingness to repeat being discharged on the day of surgery.

Our study is not without limitations. Given its reliance on patient-reported data, there exists a potential for recall bias. Patients were administered a survey 30 days postoperatively, presumed to encompass the timeframe wherein potential complications associated with expedited discharge are accounted for, while still maintaining proximity to the surgical intervention and discharge, thereby minimizing recall bias.

Overall, 10% of the patients were unwilling to repeat discharge on the day of surgery. To further enhance the satisfaction rate for discharge on day of surgery in the future, a closer examination of the reasons behind patients’ responses of ‘no’ is imperative. There are several potential internal and external factors contributing to why some patients do not wish to repeat discharge on the day of surgery if having a future hip or knee arthroplasty procedure. Gathering qualitative data on this aspect may thus offer limited additional insights capable of significantly influencing clinical practice. However, it is plausible that it could contribute to enhance patient selection and refining the day-case pathway. Considering that only 10% of the patients were unwilling to repeat discharge on the day of surgery, ongoing implementation of discharge on the day of surgery is deemed acceptable.

In conclusion, a total of 90% of patients discharged on the day of surgery after hip and knee arthroplasty were willing to repeat this length of time to discharge. This rate is encouraging and acceptable for ongoing implementation.

**Take home message**

- 90% of patients who were discharged on the day of surgery following hip and knee arthroplasty expressed willingness to repeat discharge on day of surgery.

- This rate is encouraging, and acceptable for ongoing implementation.

## Data Availability

The datasets generated and analyzed in the current study are not publicly available due to data protection regulations. Access to data is limited to the researchers who have obtained permission for data processing. Further inquiries can be made to the corresponding author.
